# Multidrug-resistant bacterial infections and their clinical impact at the University Teaching Hospital of Kigali in Rwanda: a retrospective descriptive-analytical study

**DOI:** 10.3389/fpubh.2025.1701316

**Published:** 2026-01-06

**Authors:** Jean Bosco Munyemana, Nadine Nyishimente, Samuel Rutare, Aline Nishimwe, Yves Kundwa, Daniel Manirakiza, Théoneste Nkubana, Angelique Dusabe, Francois Xavier Ndayambaje

**Affiliations:** 1 Department of Microbiology and Parasitology, School of Medicine and Pharmacy, College of Medicine and Health Sciences, University of Rwanda, Kigali, Rwanda; 2 Department of Pathology, University Teaching Hospital of Kigali, Kigali, Rwanda; 3 Department of Internal Medicine, School of Medicine and Pharmacy, College of Medicine and Health Sciences, University of Rwanda, Kigali, Rwanda; 4 Department of Pediatrics, University Teaching Hospital of Kigali, Kigali, Rwanda

**Keywords:** antimicrobial resistance, CRE, ESBL, length of hospital stay, MRSA, multidrug-resistant

## Abstract

**Introduction:**

Multidrug-resistant (MDR) bacterial infections pose a serious global health threat, particularly in low-resource settings where empirical antimicrobial use is common, while associated with poor outcomes and increased resistance. This study evaluated the prevalence, resistance profiles, clinical impact, and treatment options for MDR bacterial infections at the University Teaching Hospital of Kigali, Rwanda.

**Methods:**

A retrospective descriptive-analytical study reviewed patient records and microbiology logbooks for culture-confirmed MDR infections from 1^st^ January to 31^st^ December 2023. Data were analyzed using SPSS, with significance set at *p* < 0.05.

**Results:**

Out of 1,676 positive cultures, 368 (22%) were MDR cases, mostly from surgical patients (30.4%). Urine samples yielded the majority of MDR isolates (52.2%), with *Escherichia coli* as the most common (45.4%), particularly in urine (71.3%). MDR isolates showed high resistance rates to ampicillin, doxycycline (100%), third-generation cephalosporins (98%), amoxicillin-clavulanic acid (96%), clindamycin (88%), and ciprofloxacin (74%). Resistance was lowest against amikacin (6%), vancomycin (14%), imipenem (24%), and polymyxin B (26%). The mean hospital stay was 8.6 days, and the mortality rate was 22% among patients with MDR bacterial infection.

**Conclusion:**

MDR bacterial infections were prevalent with longer hospital stays and poor outcomes. Despite high resistance to common antibiotics, amikacin, vancomycin, imipenem, and polymyxin B were effective treatment options. Continuous surveillance, antimicrobial stewardship, and treatment guideline development are crucial.

**Discussion:**

The global rise in antimicrobial resistance is a major public health threat requiring local surveillance for targeted interventions and guideline creation. This study at the University Teaching Hospital of Kigali found a 21.9% prevalence of MDR infections, reflecting global trends. Strengthening infection prevention and control and antimicrobial stewardship programs is needed to break transmission chains and optimize antimicrobial use.

## 1 Introduction

Multidrug-resistant (MDR) bacterial infections pose a significant global threat and are a leading cause of death around the world. In 2021, there were an estimated 4.71 million deaths associated with bacterial antimicrobial resistance (AMR), including 1.14 million deaths attributable to bacterial AMR, globally (1, 2). This alarming trend is particularly evident and exhibits a huge threat in developing countries, where in 2021, the World Health Organization (WHO) African region reported over 1 million deaths associated with bacterial antimicrobial resistance, with 250,000 deaths directly attributable to MDR infections (1). However, these figures are likely underestimated due to significant gaps in surveillance and reporting systems across the African continent (3).

Different studies indicate a widespread prevalence of MDR bacterial infections with significant regional variations across Africa. In East Africa, 50–60% of urinary tract infections are caused by MDR bacteria (4), while over 80% of clinical isolates of *Acinetobacter baumannii* in South Africa are also MDR (5). In West Africa, 41.6% of MDR cases were reported from Ghana (6). The prevalence of methicillin-resistant *Staphylococcus aureus* (*MRSA*) ranges from 29 to 61% in various countries (7, 8), and Gram-negative bacteria, including *Escherichia coli, Klebsiella pneumoniae,* and *Pseudomonas aeruginosa,* show MDR phenotype rates as high as 92% in Kenyan hospitals (9, 10).

The term “multidrug resistance” refers to bacteria that are resistant to at least one agent in three or more antibiotic classes (11, 12). The genetic basis for these resistance mechanisms often involves the acquisition of plasmids carrying resistance genes, which can be transferred between bacteria, facilitating the spread of resistance traits across different species (11–13). Several factors contribute to the rising incidence of MDR, including inadequate prioritization of MDR problems, the absence of antimicrobial stewardship (AMS) programs, limited knowledge and awareness, prior exposure to antibiotics, insufficient microbiology laboratory capacity, inability to isolate patients with MDR pathogens, and non-adherence to infection prevention and control (IPC) precautions (14, 15).

MDR infections are associated with increased morbidity and mortality and prolonged hospitalization, which translates to a significant burden on healthcare systems (16). The WHO priority pathogens known as ESKAPE (*Enterococcus faecium*, *Staphylococcus aureus*, *Klebsiella pneumoniae*, *Acinetobacter baumannii*, *Pseudomonas aeruginosa*, and *Enterobacter* spp.) were reported to be major contributors responsible for MDR infections and are associated with a high mortality risk (17, 18). These pathogens not only pose a threat in hospital settings, but also contribute to substantial economic burdens due to increased healthcare costs (19, 20), while long hospitalizations, surgical procedures, mechanical ventilation, and the use of indwelling catheters have been identified as independent risk factors associated with MDR infections and poor outcomes (21).

In Rwanda, health care systems lack updated information concerning the prevalence of MDR bacterial infections. A previous study conducted at the University Teaching Hospital of Kigali in Rwanda found that gram-negative bacteria comprised 88.7% of all isolates, and among the Gram-negative isolates tested, 75.9% were resistant to ceftriaxone, 71.7% exhibited an extended-spectrum beta-lactamase (ESBL) positive phenotype, and resistance rates were 97.6% for amoxicillin-clavulanic acid and 56% for ciprofloxacin (22). Another study conducted in Rwanda revealed a high prevalence of antimicrobial resistance among commonly used antimicrobials (23). Moreover, a recent report highlights the high prevalence of ESBL producers in Rwanda, which was associated with prolonged hospital stays and a higher mortality rate in patients with infections due to ESBL producers, more than double the general hospital mortality rate in the same period (24).

These findings, together with the lack of AMR surveillance, high rates of empirical therapy, inadequate IPC standards, and lack of treatment guidelines, alarmingly call for special focus, continuous surveillance, and intervention (25). Our study intended to characterize MDR bacterial infections, assessing their prevalence, antimicrobial resistance profiles, and clinical impact, and to determine potential therapeutic options within the local context.

## 2 Methods

### 2.1 Study site

This study was conducted at the University Teaching Hospital of Kigali, also known as Centre Hospitalier Universitaire de Kigali (CHUK), the largest referral hospital in the country, with a capacity of 500 beds, approximately 16,000 to 18,000 annual admissions, and a staff of around 1200 personnel, providing care to a population estimated at around 1,000,000 from different parts of the country.

### 2.2 Study design and period

This was a retrospective descriptive-analytical study that involved a review of patient medical records, microbiology culture logbooks, and Open Clinic database entries. The study specifically focused on patients with culture-confirmed multidrug-resistant (MDR) bacterial infections recorded between January 1, 2023, and December 31, 2023.

### 2.3 Study population

The study population in this project was all clinical and culture-confirmed bacterial infections from patients who visited the key hospital departments, including internal medicine, pediatrics, intensive care unit (ICU), surgery, obstetrics-gynecology (OB-GYN), accident and emergency (A&E), and outpatient department (OPD). The outpatients were defined as those receiving care without hospital admission, typically assessed and treated on the day of presentation at CHUK and returned home the same day.

### 2.4 Inclusion criteria

This study included patients of all ages with clinical and culture-confirmed MDR bacterial infections from any sample type collected during the specified study period. Both inpatient and outpatient cases were considered for statistical analysis to provide a comprehensive overview of MDR infections across the healthcare setting. Only patients with culture-positive results that fulfilled both clinical and laboratory criteria for infection were included.

### 2.5 Exclusion criteria

Patients with negative microbiological cultures or those with positive cultures but isolates sensitive to tested antimicrobials were excluded from the data analysis. Additionally, patients whose isolates indicated colonization or asymptomatic carriage, such as asymptomatic bacteriuria, were also excluded to ensure the study focused on clinically significant infections.

### 2.6 Laboratory isolation and identification of bacteria

All samples, including urine, blood, pus, swabs, and body fluids, were cultured following the standard operating procedures (SOPs) at CHUK. Briefly, urine samples, after wet mount examination, were cultured on cysteine lactose electrolyte-deficient (CLED) agar (Oxoid, Thermo Fisher Scientific). Colonies were counted to determine significant bacterial growth, with a threshold of ≥ 10^5 colony-forming units (CFU) per milliliter typically used to confirm clinically relevant infection. This cutoff helps distinguish true infection from contamination or colonization. Further, the species identification and antimicrobial susceptibility testing were performed. For blood culture, bottles were directly incubated at 37 °C in BACTEC, an automated blood culture machine (BD, Bactec, Franklin Lakes, NJ). The subculture of positive bottles was done on sheep blood agar (SBA) (Oxoid, Thermo Fisher Scientific, UK) and incubated overnight at 37 °C, in a 5% CO_2_ incubator. Other samples were cultured on blood agar and MacConkey agar at the same time.

Bacterial identification was performed manually, beginning with Gram staining to differentiate Gram-positive cocci from Gram-negative bacilli based on color and morphology. Colony morphology was assessed alongside a panel of biochemical tests known as the “small gallery,” including triple sugar iron (TSI) (Oxoid, Thermo Fisher Scientific, UK), motility, indole, urease (MIU) (Oxoid, Thermo Fisher Scientific, UK), and citrate utilization. Gram-positive isolates were identified by their characteristic purple cocci morphology on Gram stain, indicative of a thick peptidoglycan layer. Further differentiation relied on colony morphology, hemolysis pattern, and pigmentation on blood agar.

Species-level identification employed biochemical tests such as catalase and coagulase for *Staphylococcus aureus*, esculin hydrolysis producing black precipitate for *Enterococcus* spp., bacitracin sensitivity for *Group A Streptococci*, and optochin sensitivity for *Streptococcus pneumoniae*. Gram-negative isolates demonstrated pink staining and rod-shaped morphology on Gram stain. Lactose fermentation was assessed on MacConkey agar (Oxoid, Thermo Fisher Scientific, UK), distinguishing lactose-fermenters like *Escherichia coli,* which also yields a positive indole test, from citrate-positive *Klebsiella pneumoniae*. *Pseudomonas aeruginosa* was identified by oxidase positivity, non-fermentation of sugars, and characteristic pigment production. *Acinetobacter* spp. were characterized by non-fermentation of sugars, oxidase negativity, and typical growth patterns on selective media. This combination of selective culture, microscopy, and focused biochemical testing provided robust and reliable identification of clinically relevant bacterial species. (Oxoid, Thermo Fisher Scientific, UK).

### 2.7 Antimicrobial susceptibility testing

The disk diffusion method was employed with various antibiotic disks: amikacin, 30 μg; ampicillin,10 μg; ceftazidime, 30 μg; cefotaxime, 30 μg; cefoxitin,30 μg; cefuroxime, 30 μg; ciprofloxacin,5 μg; fosfomycin, 50 μg; imipenem, 10 μg; meropenem, 10 μg; gentamicin, 10 μg; trimethoprim/sulfamethoxazole, 1.25/23.75 μg; amoxicillin-clavulanic acid, 20/10 μg; nitrofurantoin,300 μg; chloramphenicol,30 μg; piperacillin, 10 μg; piperacillin tazobactam, 30/6 μg; sulbactam ampicillin, 10 /10 μg; and tigecycline, 15 μg (BD, Franklin Lakes, NJ).

The saline suspension from a pure culture plate was prepared by adding bacterial colonies into sterile saline at 0.9% until the MacFarland turbidity standard of 0.5 was attained.

The resulting suspension was inoculated on Mueller-Hinton agar using a sterile cotton swab. After this procedure, the antibiotic disks were added to the plate with at least 25 mm between each disk and subsequently incubated at 37 °C for 18–24 h. Interpretation of inhibition zone diameters followed Clinical and Laboratory Standards Institute (CLSI) breakpoints (version 2023) (26). ESBL phenotypes were determined according to CLSI guidelines, observing the synergistic phenomenon between two cephalosporins (ceftazidime and ceftriaxone or cefotaxime) and amoxicillin-clavulanic acid. Carbapenem-resistant *Enterobacteriaceae* (CRE) were recorded for *E. coli* and *Klebsiella* isolates, which were resistant to meropenem or imipenem, while methicillin-resistant *Staphylococcus aureus* (MRSA) was considered when *Staphylococcus aureus* was resistant to cefoxitin.

### 2.8 Quality control

Quality control was performed following the internal standard operating procedure (SOP), using reference strains from the American Type Culture Collection (ATCC), including *Escherichia coli* (ATCC 25922), *Klebsiella pneumoniae* (ATCC 13883), and *Staphylococcus aureus* (ATCC 25923) (Thermo Scientific LENEXA, KS 66215 USA). These standardized reference strains ensure the accuracy and reliability of laboratory testing in accordance with established quality control protocols.

### 2.9 MDR selection

Multidrug resistance is defined as resistance to at least one antimicrobial agent in three or more antimicrobial categories. In the current study, bacterial isolates were classified as MDR if they exhibited resistance to one or more antimicrobial agents in at least three specified antimicrobial classes, thereby significantly limiting treatment options (27).

For Gram-positive bacteria, MDR was identified when resistance was noted to one or more agents within three or more of the following classes: penicillin (e.g., penicillin, amoxicillin, ampicillin), macrolides (e.g., erythromycin), tetracyclines (e.g., tetracycline, doxycycline), and glycopeptides (e.g., vancomycin, teicoplanin).

For Gram-negative bacteria, MDR cases were recorded when resistance was observed to one or more agents in at least three of the following classes: penicillin (e.g., amoxicillin-clavulanic acid, sulbactam-ampicillin), cephalosporins (e.g., cefotaxime, ceftriaxone, ceftazidime, cefepime), carbapenems (e.g., imipenem, meropenem), quinolones/fluoroquinolones (e.g., ciprofloxacin, levofloxacin), aminoglycosides (e.g., amikacin, gentamicin, tobramycin), sulfonamides and trimethoprim, and polymyxins (e.g., colistin, polymyxin B).

### 2.10 Data collection

All positive microbiology cultures were reviewed and recorded in an Excel sheet, and then cases of multidrug resistance (MDR) were filtered for further analysis. MDR was defined as an isolate resistant to at least one antimicrobial agent in three or more different antimicrobial categories (11). Clinical patient data collected included demographics (age, sex), clinical diagnosis, length of hospital stay (time of discharge minus time of admission), and patient outcomes such as discharge status and mortality. The day of outcome determination was defined as the date of hospital discharge or death during the index admission. For patients not hospitalized, outcome was assessed 30 days post-diagnosis (D30) when follow-up data were available.

### 2.11 Statistical analysis

Data were recorded into a Microsoft Excel spreadsheet and were transferred to SPSS version 27 for statistical analysis. To summarize the obtained data, descriptive statistics were used by frequencies and percentage calculations, and the student’s t-test was employed to compare the mean LHS in patients with MDR bacterial infections and the mean LHS of the general hospital population in the same period. The graphs and tables were used for results presentation, and a *p*-value less than 0.05 was considered statistically significant.

### 2.12 Ethical consideration

Ethical approval and waiver of consent to participate were obtained from the institutional review board (IRB) of the University of Rwanda, College of Medicine and Health Sciences (CMHS), as documented in CMHS/IRB/487/2023, and from the University Teaching Hospital of Kigali, as documented in EC/CHUK/004/2024. The collected data were kept confidential, where no name appeared on the data collection sheet; rather, patients’ ID was used. The information on the data collection sheet was recorded in a computer protected with a password to avoid loss of privacy. Participants’ informed consent was waived due to the retrospective study design, as the data were collected from patients’ files, records, and registries.

## 3 Results

### 3.1 Demographic characteristics of the studied participants

In this study, a total of 1,676 patients were analyzed, of whom 368 (22.0%) had MDR bacterial infections. Most patients with MDR infections were in the 16–44 years age group, comprising 9.7% of the total patient cohort and accounting for 163 cases. The next most affected age groups were those above 60 years (6.0%, 101 cases) and 45–60 years (2.9%, 48 cases). Pediatric groups, including neonates (0–28 days), infants (29 days to 12 months), and children under 5 years old, accounted for smaller proportions of MDR cases, ranging from 0.6 to 1.1%. Regarding sex distribution, males had a slightly higher proportion of MDR infections (54%) compared to females (46%) (Table 1).

**Table 1:** Demographic characteristics.

Variables		All patient (*N* = 1,676)		MDR (*n* = 368)	
		Frequency	%	Frequency	%
Age group	0–28 days (neonate)	67	4.0	14	0.8
	29 days −12 months (1 year)	118	7.0	18	1.1
	12–59 months (<5 years)	54	3.2	10	0.6
	5–15 years	91	5.4	14	0.8
	16–44 years	726	43.3	163	9.7
	45–60 years	238	14.2	48	2.9
	Above 60 years	382	22.8	101	6.0
Sex	Female	827	49.3	169	46
	Male	849	50.7	199	54

Data are presented as frequency and % unless otherwise indicated. N: number of total cases (sample size).

### 3.2 Frequency of MDR cases in different departments

**Figure 1:**
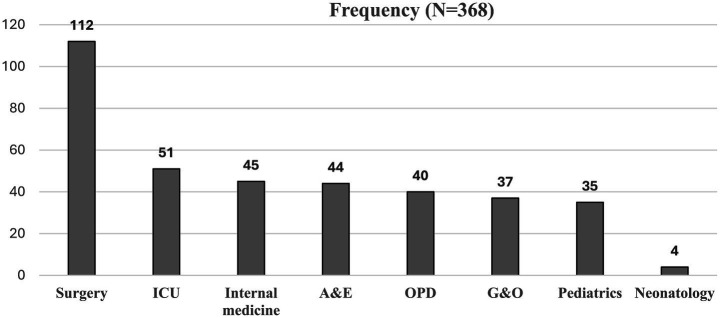
Frequency of MDR cases in different departments: Data are presented as frequency. ICU, Intensive care unit; A&E, Accident and Emergency; Outpatient Department; G&O, Gynecology and Obstetrics; N, sample size.

The distribution of MDR cases across various departments reveals significant trends in infection prevalence. The surgery department reported the highest frequency of MDR isolates with 112 (30.4%), followed by the ICU with 51 (13.8%). Other notable contributions came from internal medicine with 45 (12.2%), the accident & emergency (A&E) department with 44 (11.9%), and the outpatient department (OPD) with 40 (10.8%). Departments such as gynecology & obstetrics (G&O) and pediatrics had lower frequencies of MDR cases with 37 (10%) and 35 (9.5%), respectively, while neonatology had the least with 4 (1%) (Figure 1).

### 3.3 Distribution of MDR bacterial isolates across various sample types

The Supplementary Table S1 highlights the distribution of MDR bacterial isolates across various sample types. *E. coli* was the most prevalent isolate, accounting for 167 (45.4%) of the total 368 isolates, with a significant portion of 119/167 (71.2%) found in urine samples. *Klebsiella pneumoniae* was the second most common, representing 95 (25.8%) of the isolates, also with a notable presence in urine at 45/95 (47.3%). Urine samples had the highest total number of MDR bacterial isolates with 192 (52.2%), followed by pus with 76 isolates (20.7%), expectorate with 46 isolates (12.5%), and blood with 39 isolates (10.6%) as presented in the Supplementary Table S1.

### 3.4 Antimicrobial resistance rate in MDR isolates

The antimicrobial susceptibility patterns in MDR bacterial isolates were evaluated, revealing significant variations in resistance rates. MDR isolates were 100% resistant to ampicillin and doxycycline. Resistance rates to the third-generation cephalosporins, including cefotaxime, ceftriaxone, and ceftazidime, were 98, 98, and 95%, respectively, and 96% to amoxicillin-clavulanic acid. The resistance rate among the fluoroquinolones, including ciprofloxacin, was 74%, while the resistance rate to carbapenems such as imipenem was 24%. The resistance rate of the isolates to polymyxin B was 26%. Notably, the isolates exhibited the lowest resistance rates of 6% to amikacin and 14% to vancomycin, primarily against Gram-negative and Gram-positive bacteria, respectively (Table 2). The major bacterial isolates and their antimicrobial susceptibility patterns for some antimicrobials with higher resistance rates are presented in Supplementary Table S2.

**Table 2:** Antimicrobial resistance rate in MDR isolates.

Antibiotics	*N*	Resistance rate (%)
Ampicillin	51	100
Doxycycline	8	100
Cefotaxime	195	98
Cefuroxime	87	98
Ceftriaxone	211	98
Amoxicillin clavulanic acid	336	96
Ceftazidime	117	95
Clindamycin	17	88
Penicillin	17	88
Oxacillin/Cefoxitin	17	88
Tetracycline	8	88
Cefepime	56	79
Erythromycin	23	78
Ciprofloxacin	152	74
Piperacillin tazobactam	96	73
Gentamycin	121	70
Chloramphenicol	131	57
Nitrofurantoin	43	51
Polymyxin B	221	26
Fosfomycin	27	26
Imipenem	221	24
Vancomycin	22	14
Amikacin	181	6

Data are presented as frequency and % unless otherwise indicated. N: number of isolates tested to each antibiotic or sample size.

### 3.5 The rate of resistance phenotypes among MDR cases

**Figure 2:**
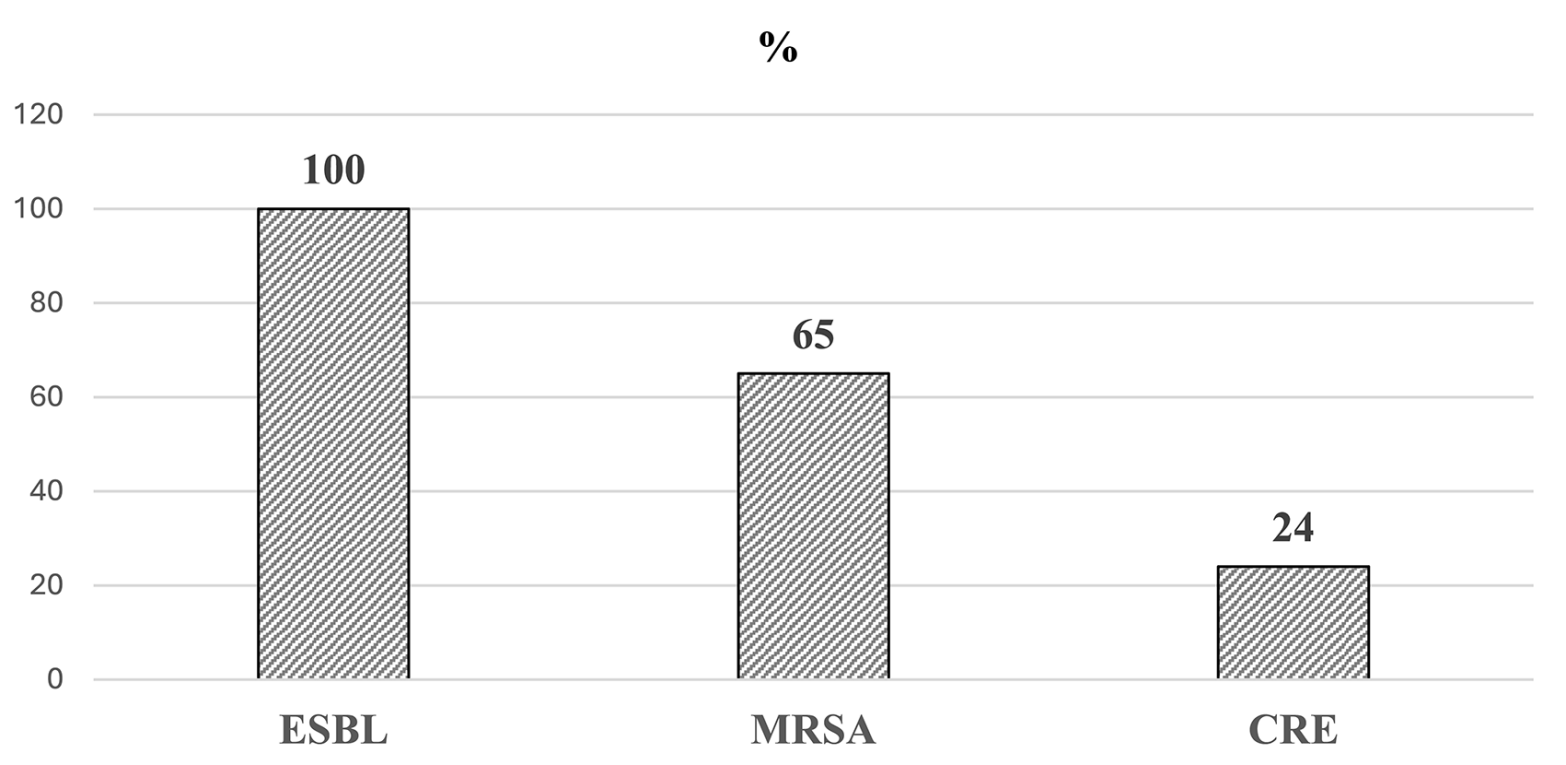
The rate of resistance phenotypes in MDR isolates: the data are presented as a percentage (%). ESBL, Extended-spectrum beta-lactamase. MRSA: Methicillin-resistant *Staphylococcus aureus*. CRE, Carbapenem-resistant *Enterobacteriaceae.*

The resistance phenotypes among 368 cases of MDR bacterial isolates were analyzed. The results indicate that Extended-Spectrum Beta-Lactamase (ESBL) producers were identified in 262 out of 262 (100%) evaluated *Klebsiella pneumoniae* and *E. coli* isolates. *Methicillin-Resistant Staphylococcus aureus* (*MRSA*) phenotypes also exhibited a notable resistance rate, with 15 out of 23 *Staphylococcus aureus* isolates (65%) displaying this characteristic. In contrast, the prevalence of *carbapenem-resistant Enterobacteriaceae* (CRE) was considerably lower, with only 52 cases identified out of 221 isolates, yielding a 24% resistance rate. Taken together, *ESBL* and *MRSA* were the more frequent resistance phenotypes among MDR cases (Figure 2).

### 3.6 Clinical impact of MDR bacterial infections

We further analysed the LHS and outcomes in patients with MDR bacterial infections. Among 153 patients for whom complete information regarding LHS and outcomes was available, most of them had a standard hospital stay of 3–14 days, with 130 out of 153 admissions (85.0%) falling within this range. Only small proportions experienced short stays of 0–2 days (12/153, 7.8%) or prolonged stays beyond 14 days (11/153, 7.2%). The average length of stay was 8.6 days, with a median of 8.0 days and a relatively wide standard deviation of 5.6 days, indicating some variation in hospitalization duration. Regarding outcomes for the 286 patients evaluated, the majority were cured (223/286, 78.0%), while 63 patients died, corresponding to a mortality rate of 22.0% (see Table 3).

**Table 3:** Hospital stays and clinical outcomes.

Variables		Frequency	%
	Short stay (0–2 days)	12	7.8
Length of hospital stays (*n* = 153)	Standard stay (3–14 Days)	130	85.0
	Long stay (>14 days)	11	7.2
	Mean/Medan	8.6/8.0	
	SD	5.6	
Outcomes (*n* = 286)	Cured	223	78.0
	Died	63	22.0

LHS, Length of hospital stay; N, sample size. Data are presented as frequency (%) unless otherwise indicated.

## 4 Discussion

The global rise of antimicrobial resistance poses a significant threat to public health, demanding local surveillance to inform targeted interventions and local antimicrobial treatment guideline development. This study has evaluated MDR bacterial culture results and clinical implications at the University Teaching Hospital of Kigali over a period of 1 year. Our findings, indicating a 21.9% overall MDR prevalence, with 100% ESBL, 65% MRSA, and 24% CRE, reflect global trends in antimicrobial resistance, particularly the significant burden of MRSA, which aligns with international data, such as a study from Nepal where 60.6% of *Staphylococcus aureus* isolates were MRSA. Despite regional variations, these figures underscore the widespread concern regarding MDR pathogens worldwide. The 2022 Global Antimicrobial Resistance and Use Surveillance System (GLASS) report highlighted median resistance rates of 42% for third-generation cephalosporin-resistant *E. coli* and 35% for MRSA across 76 countries (28). This is consistent with our previous work on ESBL producers, where we reported a prevalence of 23.4% at the same institution in 2022 (24).

This high prevalence of MDR bacterial infections could be linked to different factors, including antibiotic misuse such as self-medication and incomplete treatment, and inappropriate prescriptions, which promote the emergence of resistant strains. Additionally, multidrug resistance may also be linked to a lack of diagnostic capacity, higher rates of empirical therapy, use of leftover antibiotics, absence of standardized surveillance systems, and lack of antibiotic treatment guidelines. However, the prevalence of MDR bacteria varies significantly by region, influenced by local antibiotic prescribing practices and infection control measures (6, 29).

In the current study, MDR cases were more often found in patients from the surgical department (112/368,30%) and the ICU (53/368,14%). Our findings are consistent with previous studies (30–32). Mukagendaneza et al. (33) found that MDR cases comprised half of the studied isolates, leaving clinicians with few drug choices for the treatment of patients with surgical site infections (SSI) while patients’ own pathogens were most often reported to be responsible for SSI (34). The predominance of MDR bacterial infections in surgical departments and the ICU can be attributed to several interrelated factors. Surgical procedures and invasive techniques, coupled with exposure to pathogens and poor IPC practices, increase the risk of infections (21). Patients undergoing surgery often have compromised immune systems, making them more susceptible, while the ICU environment, where invasive monitoring and treatments occur, facilitates the transmission of pathogens with a mostly MDR phenotype (35). Furthermore, antibiotic misuse and overuse, particularly in surgical and ICU settings, and carriage of MDR bacteria in individuals admitted for surgical procedures, contribute to surgical site infections and hospital spread of these strains (36, 37). These factors align and explain the predominance of MDR bacterial infections from surgical and ICU patients in our study.

In the current study, *E. coli* was the most prevalent isolate, accounting for 45.4% of the total isolates and mostly isolated from urine samples. Additionally, we found that *K. pneumoniae* was the second most common, representing 25.8% of the isolates, with a notable presence in urine samples. This is consistent with a previous report, which found a high prevalence of *E. coli* (64.67%) and *K. pneumoniae* (35.33%) among ESBL-positive cases (24). This also aligns with findings from other studies in Tanzania (38) and Rwanda (23, 39). The higher prevalence in urine samples further underscores the significance of these isolates in urinary tract infections (40).

Moreover, this study has evaluated the antimicrobial resistance profiles of MDR isolates and found very high resistance rates to commonly used antibiotics, including third-generation cephalosporins. Interestingly, vancomycin (for Gram-positive isolates), amikacin, imipenem, fosfomycin, and polymyxin B (for Gram-negative isolates) exhibited lower resistance compared to the other antibiotics tested. Similar findings have been reported (2, 23, 39), and these antibiotics with lower resistance rates could be alternatively suggested as potential drugs for MDR bacterial infections and can provide guidance for antimicrobial treatment guideline development within the local context. However, precautions should be taken to prevent further resistance development.

In our study, ESBL (100%) and MRSA (65%) phenotypes were the most prevalent, while CRE (19.8%) was the least prevalent. This is similar to our previous report on the prevalence of ESBL-producing Enterobacteriaceae at the University Teaching Hospital of Kigali in Rwanda in 2022, which showed a prevalence of 23.4% (24), and other studies on MRSA in Nigeria (7), Cameroon (8), and Nepal (41). However, this study focused only on MDR cases, whereby all *E. coli* and *K. pneumoniae* isolates were 100% ESBL producers. The higher resistance to third-generation cephalosporins and ESBL production highlights accumulated overuse and misuse of these antimicrobials, as previously reported (42). Unfortunately, further resistance acquisition is expected if no action is taken today, as pathogens acquire new resistance mostly by plasmid transfer or intrinsically after exposure to lower concentrations of antimicrobials (43).

In our study population, the mean hospital stay was 8.6 days, and the mortality rate was 22% among patients with MDR bacterial infection, both notably higher than CHUK’s general average stay of 6 days and mortality rate of 5.8% in 2023. These data underscore a significantly elevated mortality in MDR-infected patients relative to the wider hospital population, highlighting the substantial impact of multidrug resistance on patient outcomes at CHUK. These prolonged hospitalizations and elevated mortality rates align with previous findings, underscoring the substantial impact of MDR infections on patient outcomes and healthcare resources (24, 25).

The patients with MDR bacterial infections are vulnerable, and prognosis is poor if appropriate and susceptible antimicrobials are not administered promptly. An estimated 700,000 deaths occur globally every year as a result of infections caused by antimicrobial-resistant pathogens, and AMR also contributes directly to the decline in the economy (19), where sub-Saharan Africa suffers the most with the highest mortality rate (23.5 deaths per 100,000) attributable to AMR compared to other regions (44). This problem of resistance alarmingly calls for intervention and collaborative effort for improving patients’ health outcomes.

To address this global crisis, several interventions have been suggested and reported to be associated with improved clinical outcomes. A well-coordinated One Health approach, which is a collaborative effort across multiple disciplines including human health, animal health, and agriculture, aims to optimize antimicrobial use (45). Another important aspect is the antimicrobial stewardship (AMS) program, which promotes best practices and optimizes antimicrobial use (46). An AMS program has proven to be an important pillar in reducing antibiotic use and surgical antibiotic prophylaxis misuse, correlating with improved patient health outcomes (47). Combining both the One Health approach and a well-coordinated antimicrobial stewardship could help address the crisis and contribute to future lifesaving efforts in developing countries and beyond.

Our study has several limitations, including a one-year surveillance period that may not capture the full scope of MDR bacterial infections and incomplete patient data, especially regarding outcomes and discharge times. Additionally, overall hospital mortality and length of stay were reported without adjusting for differences in patient populations or infection attribution, limiting causal inference. Despite these constraints, our findings provide valuable insights to raise awareness of MDR in Rwanda and assist policymakers in developing targeted interventions and antimicrobial treatment guidelines.

## 5 Conclusion

This study reveals a higher prevalence of MDR bacterial infections with poor outcomes, alongside significant resistance to commonly used antimicrobials. However, vancomycin, meropenem, amikacin, and polymyxin B showed the least resistance, offering potential treatment options for MDR bacterial infections. The findings underscore the urgent need for continued surveillance and reinforcement of antimicrobial stewardship programs to monitor resistance patterns and maintain the effectiveness of these critical antimicrobial agents.

## Data Availability

The original contributions presented in the study are included in the article/Supplementary material, further inquiries can be directed to the corresponding author.
