# Supplementary material for: Multidrug-resistant bacterial infections and their clinical impact at the University Teaching Hospital of Kigali in Rwanda: a retrospective descriptive-analytical study
Source: Front Public Health. 2026 Jan 6;13:1701316. doi: 10.3389/fpubh.2025.1701316 (PMC12815734; doi:10.3389/fpubh.2025.1701316)
Supplement: Supplementary file 1 [file Table_1.DOCX]

**Supplementary Table 1: Distribution of MDR Bacterial isolates according to sample types**

| **Bacterial isolates** | **Blood** | **Expectorate** | **Urethral swabs** | **Vaginal swabs** | **Gastric aspirates** | **Pus** | **Tracheal aspirates** | **Urine** | **Total** |
| --- | --- | --- | --- | --- | --- | --- | --- | --- | --- |
| ***Acinetobacter baumannii*** | **3** | **17** | **0** | **0** | **0** | **8** | **3** | **6** | **37(10.1)** |
| *Citrobacter species* | 2 | 0 | 0 | 0 | 0 | 0 | 0 | 0 | 2(0.5) |
| *Enterobacter cloacae* | 2 | 0 | 0 | 0 | 0 | 0 | 0 | 0 | 2(0.5) |
| ***Escherichia coli*** | **6** | **14** | **0** | **0** | **0** | **27** | **1** | **119** | **167(45.4)** |
| *Enterobacter species* | 1 | 0 | 0 | 0 | 0 | 3 | 0 | 6 | 10(2.7) |
| *Enterococcus species,* | 0 | 0 | 0 | 0 | 0 | 1 | 0 | 0 | 1(0.3) |
| *Klebsiella oxytoca* | 1 | 0 | 0 | 0 | 1 | 4 | 0 | 6 | 12(3.3) |
| ***Klebsiella pneumoniae*** | **14** | **10** | **0** | **0** | **1** | **21** | **4** | **45** | **95(25.8)** |
| *Pseudomonas aeruginosa* | 0 | 2 | 0 | 0 | 0 | 0 | 1 | 1 | 4(1.1) |
| *Proteus species* | 0 | 3 | 1 | 0 | 0 | 4 | 0 | 4 | 12(3.3) |
| *Providencia species* | 0 | 0 | 0 | 0 | 0 | 1 | 0 | 0 | 1(0.3) |
| ***Staphylococcus aureus*** | **9** | **0** | **0** | **3** | **0** | **8** | **0** | **3** | **23(6.3)** |
| *Stenotrophomonas maltophilia* | 0 | 0 | 0 | 0 | 0 | 0 | 0 | 1 | 1(0.3) |
| *Streptococcus species* | 1 | 0 | 0 | 0 | 0 | 0 | 0 | 0 | 1(0.3) |
| **Total** | 39(10.6) | 46(12.5) | 1(0.3) | 3(0.8) | 2(0.5) | 76(20.7) | 9(2.4) | **192(52.2)** | 368(100) |

Data are presented as frequency (%) unless otherwise indicated.

**Supplementary Table 2: Antimicrobial susceptibility testing results for key isolates on selected antimicrobials**

| **No** | **Sample type** | **Culture results** | **AMC** | **AMP** | **CAZ** | **CRO** | **CTX** | **CXM** | **DO** |
| --- | --- | --- | --- | --- | --- | --- | --- | --- | --- |
| 1 | Blood | *Acinetobacter baumannii* | R |  |  |  |  |  |  |
| 2 | Blood | *Acinetobacter baumannii* | R |  | R |  |  |  |  |
| 3 | Blood | *Acinetobacter baumannii* | R |  |  |  |  |  |  |
| 4 | Expectorate | *Acinetobacter baumannii* | R |  | R | R | R |  |  |
| 5 | Expectorate | *Acinetobacter baumannii* | R |  |  |  |  | R |  |
| 6 | Expectorate | *Acinetobacter baumannii* | R |  |  | R | R |  |  |
| 7 | Expectorate | *Acinetobacter baumannii* | R |  |  |  |  |  |  |
| 8 | Expectorate | *Acinetobacter baumannii* | R |  |  |  | R |  |  |
| 9 | Expectorate | *Acinetobacter baumannii* | R |  |  |  | R |  |  |
| 10 | Expectorate | *Acinetobacter baumannii* | R |  |  | R |  |  |  |
| 11 | Expectorate | *Acinetobacter baumannii* | R |  | S |  | R | R |  |
| 12 | Expectorate | *Acinetobacter baumannii* | R |  | R |  | R |  |  |
| 13 | Expectorate | *Acinetobacter baumannii* | R |  | R | R | R |  |  |
| 14 | Expectorate | *Acinetobacter baumannii* | R |  | R |  |  |  |  |
| 15 | Expectorate | *Acinetobacter baumannii* | R |  | R | R | R |  |  |
| 16 | Expectorate | *Acinetobacter baumannii* | R |  | R |  | R |  |  |
| 17 | Expectorate | *Acinetobacter baumannii* | R |  | R | R | R |  |  |
| 18 | Expectorate | *Acinetobacter baumannii* | R |  | R |  |  |  |  |
| 19 | Expectorate | *Acinetobacter baumannii* | R |  |  | R | R |  |  |
| 20 | Expectorate | *Acinetobacter baumannii* | R |  |  |  |  |  |  |
| 21 | Pus | *Acinetobacter baumannii* |  |  | S |  |  |  |  |
| 22 | Pus | *Acinetobacter baumannii* | R |  |  | R |  |  |  |
| 23 | Pus | *Acinetobacter baumannii* | R |  |  |  | R | R |  |
| 24 | Pus | *Acinetobacter baumannii* | R |  |  |  | R |  |  |
| 25 | Pus | *Acinetobacter baumannii* | R |  |  |  | R | R |  |
| 26 | Pus | *Acinetobacter baumannii* | R |  | R | R | R |  |  |
| 27 | Pus | *Acinetobacter baumannii* | R |  | R | R | R |  |  |
| 28 | Pus | *Acinetobacter baumannii* | R |  |  |  |  |  |  |
| 29 | Tracheal aspirates | *Acinetobacter baumannii* | R |  | R |  |  |  |  |
| 30 | Tracheal aspirates | *Acinetobacter baumannii* | R |  |  |  |  |  |  |
| 31 | Tracheal aspirates | *Acinetobacter baumannii* |  |  | R |  |  | R |  |
| 32 | Urine | *Acinetobacter baumannii* | R |  |  | R |  |  |  |
| 33 | Urine | *Acinetobacter baumannii* |  |  |  | R |  |  |  |
| 34 | Urine | *Acinetobacter baumannii* | R |  |  | R |  | R |  |
| 35 | Urine | *Acinetobacter baumannii* | R |  |  |  | R | R |  |
| 36 | Urine | *Acinetobacter baumannii* | R |  |  | R | R |  |  |
| 37 | Urine | *Acinetobacter baumannii* | R |  |  | R | R |  |  |
| 38 | Blood | *Escherichia coli* | R |  | R |  | R |  |  |
| 39 | Blood | *Escherichia coli* | R |  | R |  | R |  |  |
| 40 | Blood | *Escherichia coli* | R | R |  | R |  | R |  |
| 41 | Blood | *Escherichia coli* | S |  |  |  | R |  |  |
| 42 | Blood | *Escherichia coli* | R |  |  | R | R |  |  |
| 43 | Blood | *Escherichia coli* | R |  |  | R |  |  |  |
| 44 | Expectorate | *Escherichia coli* | R |  | R |  | R |  |  |
| 45 | Expectorate | *Escherichia coli* | R | R |  | R |  |  |  |
| 46 | Expectorate | *Escherichia coli* | R |  |  | R | R |  |  |
| 47 | Expectorate | *Escherichia coli* | R |  |  | R | R |  |  |
| 48 | Expectorate | *Escherichia coli* | R |  |  | R | R |  |  |
| 49 | Expectorate | *Escherichia coli* | R |  |  | R |  |  |  |
| 50 | Expectorate | *Escherichia coli* | R |  |  | R | R |  |  |
| 51 | Expectorate | *Escherichia coli* | R |  |  | R | R |  |  |
| 52 | Expectorate | *Escherichia coli* | R |  |  | R |  |  |  |
| 53 | Expectorate | *Escherichia coli* | R |  |  | R |  |  |  |
| 54 | Expectorate | *Escherichia coli* | R |  | R | R | R |  |  |
| 55 | Expectorate | *Escherichia coli* | R |  | R | R |  |  |  |
| 56 | Expectorate | *Escherichia coli* | R |  | R |  | R | R |  |
| 57 | Expectorate | *Escherichia coli* | R |  | R |  | R |  |  |
| 58 | Pus | *Escherichia coli* | R |  |  | R | R |  |  |
| 59 | Pus | *Escherichia coli* | R |  |  |  | R |  |  |
| 60 | Pus | *Escherichia coli* | R |  |  | R | R |  |  |
| 61 | Pus | *Escherichia coli* | R |  |  | R | R |  |  |
| 62 | Pus | *Escherichia coli* | R |  |  | R |  |  |  |
| 63 | Pus | *Escherichia coli* | R |  |  | R |  |  |  |
| 64 | Pus | *Escherichia coli* | R |  |  | R |  |  |  |
| 65 | Pus | *Escherichia coli* | R |  |  | R |  |  |  |
| 66 | Pus | *Escherichia coli* | R |  |  | R | R |  |  |
| 67 | Pus | *Escherichia coli* | R |  |  | R | R |  |  |
| 68 | Pus | *Escherichia coli* | R |  |  | R | R |  |  |
| 69 | Pus | *Escherichia coli* | R |  |  | R |  |  |  |
| 70 | Pus | *Escherichia coli* | R |  |  |  | R | R |  |
| 71 | Pus | *Escherichia coli* | R |  |  | R |  |  |  |
| 72 | Pus | *Escherichia coli* | R |  |  |  | R |  |  |
| 73 | Pus | *Escherichia coli* | R |  |  | R |  |  |  |
| 74 | Pus | *Escherichia coli* | R |  |  | R |  |  |  |
| 75 | Pus | *Escherichia coli* | R |  | R |  |  |  |  |
| 76 | Pus | *Escherichia coli* | R |  | R | R |  | R |  |
| 77 | Pus | *Escherichia coli* | R |  | R | S | S |  |  |
| 78 | Pus | *Escherichia coli* | R |  | R | R | R |  |  |
| 79 | Pus | *Escherichia coli* | R |  | R |  | R |  |  |
| 80 | Pus | *Escherichia coli* | R |  | R |  | R |  |  |
| 81 | Pus | *Escherichia coli* | R |  |  |  | R |  |  |
| 82 | Pus | *Escherichia coli* | R |  | R |  | R |  |  |
| 83 | Pus | *Escherichia coli* | S | R |  | R |  | R |  |
| 84 | Pus | *Escherichia coli* | R |  |  |  | R |  |  |
| 85 | Tracheal aspirates | *Escherichia coli* | R |  | R |  |  | R |  |
| 86 | Urine | *Escherichia coli* |  | R | R |  |  |  |  |
| 87 | Urine | *Escherichia coli* | R |  |  |  | R |  |  |
| 88 | Urine | *Escherichia coli* | R |  | R | R | R |  |  |
| 89 | Urine | *Escherichia coli* | R |  | R | R |  |  |  |
| 90 | Urine | *Escherichia coli* | R |  |  | R | R |  |  |
| 91 | Urine | *Escherichia coli* | R | R |  | R | R |  |  |
| 92 | Urine | *Escherichia coli* | R | R |  | R | R |  |  |
| 93 | Urine | *Escherichia coli* | R |  |  | R | R |  |  |
| 94 | Urine | *Escherichia coli* | R | R |  | R | R |  |  |
| 95 | Urine | *Escherichia coli* | R |  |  | R |  |  |  |
| 96 | Urine | *Escherichia coli* | R | R |  | R | R |  |  |
| 97 | Urine | *Escherichia coli* | S |  |  |  | R |  |  |
| 98 | Urine | *Escherichia coli* | R |  | R | R |  |  |  |
| 99 | Urine | *Escherichia coli* | R | R |  | R | R |  |  |
| 100 | Urine | *Escherichia coli* | R |  |  | R | R |  |  |
| 101 | Urine | *Escherichia coli* | R |  |  | R |  |  |  |
| 102 | Urine | *Escherichia coli* |  |  |  | R | R | R |  |
| 103 | Urine | *Escherichia coli* | R |  |  |  | R |  |  |
| 104 | Urine | *Escherichia coli* | R |  |  |  | R |  |  |
| 105 | Urine | *Escherichia coli* | R |  |  | R |  |  |  |
| 106 | Urine | *Escherichia coli* | R |  |  | R |  |  |  |
| 107 | Urine | *Escherichia coli* | R |  |  |  | R |  |  |
| 108 | Urine | *Escherichia coli* | R |  |  | R | R |  |  |
| 109 | Urine | *Escherichia coli* | R |  |  | R | R |  |  |
| 110 | Urine | *Escherichia coli* | R |  |  | R | R |  |  |
| 111 | Urine | *Escherichia coli* | R |  |  | R | R |  |  |
| 112 | Urine | *Escherichia coli* | R |  |  | R | R | R |  |
| 113 | Urine | *Escherichia coli* | R | R |  | R | R |  |  |
| 114 | Urine | *Escherichia coli* | R |  | R | R | R |  |  |
| 115 | Urine | *Escherichia coli* | R |  |  | R | R |  |  |
| 116 | Urine | *Escherichia coli* | R |  |  | R | R |  |  |
| 117 | Urine | *Escherichia coli* | R |  |  | R | R |  |  |
| 118 | Urine | *Escherichia coli* | R |  |  | S | R |  |  |
| 119 | Urine | *Escherichia coli* | R |  |  | R |  |  |  |
| 120 | Urine | *Escherichia coli* | R |  |  |  | R | R |  |
| 121 | Urine | *Escherichia coli* | R |  |  |  |  |  |  |
| 122 | Urine | *Escherichia coli* | R |  |  |  | R |  |  |
| 123 | Urine | *Escherichia coli* | R |  |  |  | R |  |  |
| 124 | Urine | *Escherichia coli* | R |  |  |  | R |  |  |
| 125 | Urine | *Escherichia coli* | R |  |  | R | R |  |  |
| 126 | Urine | *Escherichia coli* | R |  |  | R | R | R |  |
| 127 | Urine | *Escherichia coli* | R | R |  | R | R |  |  |
| 128 | Urine | *Escherichia coli* | R |  |  | R | R | R |  |
| 129 | Urine | *Escherichia coli* | R |  |  | R | R |  |  |
| 130 | Urine | *Escherichia coli* | R |  |  | S | S |  |  |
| 131 | Urine | *Escherichia coli* | R |  | R |  |  |  |  |
| 132 | Urine | *Escherichia coli* | R |  | R |  |  | R |  |
| 133 | Urine | *Escherichia coli* | R |  |  | R | R |  |  |
| 134 | Urine | *Escherichia coli* | R |  |  | R | R |  |  |
| 135 | Urine | *Escherichia coli* | R |  |  | R | R |  |  |
| 136 | Urine | *Escherichia coli* | R |  |  | R | R |  |  |
| 137 | Urine | *Escherichia coli* | R |  |  | R | R |  |  |
| 138 | Urine | *Escherichia coli* | R |  |  |  | R |  |  |
| 139 | Urine | *Escherichia coli* | R |  |  | R |  |  |  |
| 140 | Urine | *Escherichia coli* | R |  |  | R |  |  |  |
| 141 | Urine | *Escherichia coli* | R |  |  |  |  |  |  |
| 142 | Urine | *Escherichia coli* | R |  |  |  | R |  |  |
| 143 | Urine | *Escherichia coli* | R |  |  | R |  |  |  |
| 144 | Urine | *Escherichia coli* | R |  |  | R |  |  |  |
| 145 | Urine | *Escherichia coli* | R |  |  |  | R | R |  |
| 146 | Urine | *Escherichia coli* | R |  |  | R |  |  |  |
| 147 | Urine | *Escherichia coli* | R |  |  | R |  |  |  |
| 148 | Urine | *Escherichia coli* | R |  |  |  |  | R |  |
| 149 | Urine | *Escherichia coli* | R |  |  |  | R |  |  |
| 150 | Urine | *Escherichia coli* | R |  |  | R | R |  |  |
| 151 | Urine | *Escherichia coli* | R |  |  |  | R | R |  |
| 152 | Urine | *Escherichia coli* | R |  |  | R | R |  |  |
| 153 | Urine | *Escherichia coli* | R |  |  | R | R |  |  |
| 154 | Urine | *Escherichia coli* | R |  |  | R |  |  |  |
| 155 | Urine | *Escherichia coli* | R |  |  | R | R |  |  |
| 156 | Urine | *Escherichia coli* | R |  |  | R | R |  |  |
| 157 | Urine | *Escherichia coli* | R |  | R | R |  |  |  |
| 158 | Urine | *Escherichia coli* |  |  | R |  |  |  |  |
| 159 | Urine | *Escherichia coli* | R |  |  | R |  |  |  |
| 160 | Urine | *Escherichia coli* | R |  | R |  | R |  |  |
| 161 | Urine | *Escherichia coli* | R |  | R |  |  | R |  |
| 162 | Urine | *Escherichia coli* | R |  |  | R |  | R |  |
| 163 | Urine | *Escherichia coli* | R |  | R |  | R | R |  |
| 164 | Urine | *Escherichia coli* | R |  |  | S | R |  |  |
| 165 | Urine | *Escherichia coli* | R |  |  | R |  |  |  |
| 166 | Urine | *Escherichia coli* | R |  |  | R | R | R |  |
| 167 | Urine | *Escherichia coli* | R |  | R | R | R |  |  |
| 168 | Urine | *Escherichia coli* | R |  | R | R | R |  |  |
| 169 | Urine | *Escherichia coli* | R |  |  | R | R | R |  |
| 170 | Urine | *Escherichia coli* | R |  |  | R |  |  |  |
| 171 | Urine | *Escherichia coli* | R |  |  | R |  |  |  |
| 172 | Urine | *Escherichia coli* | R |  | R | R |  |  |  |
| 173 | Urine | *Escherichia coli* | R |  | R |  |  |  |  |
| 174 | Urine | *Escherichia coli* | R |  | R |  |  |  |  |
| 175 | Urine | *Escherichia coli* | R |  | R |  | R | R |  |
| 176 | Urine | *Escherichia coli* | R |  |  | R | R |  |  |
| 177 | Urine | *Escherichia coli* | R |  | R |  | R | R |  |
| 178 | Urine | *Escherichia coli* | R |  | R | R | R | R |  |
| 179 | Urine | *Escherichia coli* | R |  | R | R |  |  |  |
| 180 | Urine | *Escherichia coli* | R |  | R | R |  |  |  |
| 181 | Urine | *Escherichia coli* | R |  | R | R |  |  |  |
| 182 | Urine | *Escherichia coli* | R |  | R | R |  |  |  |
| 183 | Urine | *Escherichia coli* | R |  | R | R |  |  |  |
| 184 | Urine | *Escherichia coli* | R |  | R |  | R | R |  |
| 185 | Urine | *Escherichia coli* | R |  | R |  | R |  |  |
| 186 | Urine | *Escherichia coli* | R |  | R |  | R |  |  |
| 187 | Urine | *Escherichia coli* | R |  | R |  | R |  |  |
| 188 | Urine | *Escherichia coli* | S |  | S |  | S |  |  |
| 189 | Urine | *Escherichia coli* | R |  | R |  | R |  |  |
| 190 | Urine | *Escherichia coli* | R | R |  |  |  | R |  |
| 191 | Urine | *Escherichia coli* | R | R | R | R |  | R |  |
| 192 | Urine | *Escherichia coli* | S | R | R | R |  | R |  |
| 193 | Urine | *Escherichia coli* | R | R |  | R |  | R |  |
| 194 | Urine | *Escherichia coli* | R |  | R |  | R |  |  |
| 195 | Urine | *Escherichia coli* | S | R |  | R |  | R |  |
| 196 | Urine | *Escherichia coli* | R | R |  | R |  |  |  |
| 197 | Urine | *Escherichia coli* | R |  | R |  | R |  |  |
| 198 | Urine | *Escherichia coli* | R |  | R |  | R |  |  |
| 199 | Urine | *Escherichia coli* | S | R |  | S |  | S |  |
| 200 | Urine | *Escherichia coli* | S | R |  | R |  | R |  |
| 201 | Urine | *Escherichia coli* | S | R |  | R |  | R |  |
| 202 | Urine | *Escherichia coli* | S | R |  | R |  | R |  |
| 203 | Urine | *Escherichia coli* | R |  | R | R | R | R |  |
| 204 | Urine | *Escherichia coli* | R | R |  | R |  | R |  |
| 205 | Blood | *Klebsiella pneumoniae* | R |  |  | R | R |  |  |
| 206 | Blood | *Klebsiella pneumoniae* | R |  | R |  | R | R |  |
| 207 | Blood | *Klebsiella pneumoniae* | R | R |  | R |  | R |  |
| 208 | Blood | *Klebsiella pneumoniae* | R | R |  | R |  | R |  |
| 209 | Blood | *Klebsiella pneumoniae* | R | R |  | R |  |  |  |
| 210 | Blood | *Klebsiella pneumoniae* | R | R |  |  |  | R |  |
| 211 | Blood | *Klebsiella pneumoniae* | R | R |  | R |  | R |  |
| 212 | Blood | *Klebsiella pneumoniae* | R |  | R |  | R |  |  |
| 213 | Blood | *Klebsiella pneumoniae* | R | R |  | R |  | R |  |
| 214 | Blood | *Klebsiella pneumoniae* | R |  |  | R | R |  |  |
| 215 | Blood | *Klebsiella pneumoniae* | R |  |  | R | R |  |  |
| 216 | Blood | *Klebsiella pneumoniae* | R |  |  |  | R |  |  |
| 217 | Blood | *Klebsiella pneumoniae* | R |  |  | R | R |  |  |
| 218 | Blood | *Klebsiella pneumoniae* | R |  | R | R |  |  |  |
| 219 | Expectorate | *Klebsiella pneumoniae* |  |  |  | R | R |  |  |
| 220 | Expectorate | *Klebsiella pneumoniae* | R |  |  | R | R |  |  |
| 221 | Expectorate | *Klebsiella pneumoniae* | R |  |  | R |  |  |  |
| 222 | Expectorate | *Klebsiella pneumoniae* | R |  |  | R |  |  |  |
| 223 | Expectorate | *Klebsiella pneumoniae* | R |  |  | R | R |  |  |
| 224 | Expectorate | *Klebsiella pneumoniae* | R |  | R |  |  | R |  |
| 225 | Expectorate | *Klebsiella pneumoniae* | R |  | R |  |  | R |  |
| 226 | Expectorate | *Klebsiella pneumoniae* | R |  | R | R |  |  |  |
| 227 | Expectorate | *Klebsiella pneumoniae* | R |  | R | R |  |  |  |
| 228 | Expectorate | *Klebsiella pneumoniae* | R |  | R |  | R |  |  |
| 229 | Gastric aspirates | *Klebsiella pneumoniae* | R | R | R | R |  | R |  |
| 230 | Pus | *Klebsiella pneumoniae* | R |  | R |  | R |  |  |
| 231 | Pus | *Klebsiella pneumoniae* | R |  |  | R | R |  |  |
| 232 | Pus | *Klebsiella pneumoniae* | R |  |  | R | R |  |  |
| 233 | Pus | *Klebsiella pneumoniae* | R | R | R |  | R |  |  |
| 234 | Pus | *Klebsiella pneumoniae* | R |  |  | R |  |  |  |
| 235 | Pus | *Klebsiella pneumoniae* | R |  |  |  |  |  |  |
| 236 | Pus | *Klebsiella pneumoniae* | R |  |  | R |  |  |  |
| 237 | Pus | *Klebsiella pneumoniae* | R |  |  | R |  |  |  |
| 238 | Pus | *Klebsiella pneumoniae* | R |  |  |  |  |  |  |
| 239 | Pus | *Klebsiella pneumoniae* | R |  |  | R |  |  |  |
| 240 | Pus | *Klebsiella pneumoniae* | R |  |  | R | R |  |  |
| 241 | Pus | *Klebsiella pneumoniae* | R |  |  |  | R | R |  |
| 242 | Pus | *Klebsiella pneumoniae* | R |  |  |  | R |  |  |
| 243 | Pus | *Klebsiella pneumoniae* | R |  |  | R | R |  |  |
| 244 | Pus | *Klebsiella pneumoniae* | R |  |  | R | R |  |  |
| 245 | Pus | *Klebsiella pneumoniae* | R |  | R | R |  |  |  |
| 246 | Pus | *Klebsiella pneumoniae* | R |  | R |  | R |  |  |
| 247 | Pus | *Klebsiella pneumoniae* | R |  | R | R | R |  |  |
| 248 | Pus | *Klebsiella pneumoniae* | R |  |  |  | R |  |  |
| 249 | Pus | *Klebsiella pneumoniae* | R |  | R |  | R |  |  |
| 250 | Pus | *Klebsiella pneumoniae* | R |  | R |  | R |  |  |
| 251 | Tracheal aspirates | *Klebsiella pneumoniae* | R |  |  |  | R | R |  |
| 252 | Tracheal aspirates | *Klebsiella pneumoniae* | R |  | R |  |  | R |  |
| 253 | Tracheal aspirates | *Klebsiella pneumoniae* | R |  | R |  |  |  |  |
| 254 | Tracheal aspirates | *Klebsiella pneumoniae* | S | R |  | R |  | R |  |
| 255 | Urine | *Klebsiella pneumoniae* |  |  | R | R | R |  |  |
| 256 | Urine | *Klebsiella pneumoniae* | R |  | R | R | R |  |  |
| 257 | Urine | *Klebsiella pneumoniae* |  |  |  | R |  |  |  |
| 258 | Urine | *Klebsiella pneumoniae* | R |  |  | R | R |  |  |
| 259 | Urine | *Klebsiella pneumoniae* |  |  |  | R |  | R |  |
| 260 | Urine | *Klebsiella pneumoniae* | R |  |  | R | R |  |  |
| 261 | Urine | *Klebsiella pneumoniae* | R |  |  | R | R |  |  |
| 262 | Urine | *Klebsiella pneumoniae* | R |  |  | R | R | R |  |
| 263 | Urine | *Klebsiella pneumoniae* | R |  |  | R | R |  |  |
| 264 | Urine | *Klebsiella pneumoniae* | R |  |  |  | R | R |  |
| 265 | Urine | *Klebsiella pneumoniae* | R |  |  | R |  |  |  |
| 266 | Urine | *Klebsiella pneumoniae* | R | R |  | R | R |  |  |
| 267 | Urine | *Klebsiella pneumoniae* | R |  |  |  | R | R |  |
| 268 | Urine | *Klebsiella pneumoniae* | R |  |  |  |  |  |  |
| 269 | Urine | *Klebsiella pneumoniae* | R |  |  |  |  |  |  |
| 270 | Urine | *Klebsiella pneumoniae* | R |  |  | R |  |  |  |
| 271 | Urine | *Klebsiella pneumoniae* | R |  |  | R | R |  |  |
| 272 | Urine | *Klebsiella pneumoniae* | R | R |  | R | R |  |  |
| 273 | Urine | *Klebsiella pneumoniae* | R |  |  |  | R |  |  |
| 274 | Urine | *Klebsiella pneumoniae* | R |  |  | R | R | R |  |
| 275 | Urine | *Klebsiella pneumoniae* | R |  |  |  |  |  |  |
| 276 | Urine | *Klebsiella pneumoniae* | R |  |  |  | R | R |  |
| 277 | Urine | *Klebsiella pneumoniae* | R |  |  | R |  | R |  |
| 278 | Urine | *Klebsiella pneumoniae* | R |  |  | R |  |  |  |
| 279 | Urine | *Klebsiella pneumoniae* | R |  |  | R |  |  |  |
| 280 | Urine | *Klebsiella pneumoniae* | R |  | R |  |  | R |  |
| 281 | Urine | *Klebsiella pneumoniae* | R |  |  | R |  |  |  |
| 282 | Urine | *Klebsiella pneumoniae* | R |  | R | R |  |  |  |
| 283 | Urine | *Klebsiella pneumoniae* | R |  | R |  |  |  |  |
| 284 | Urine | *Klebsiella pneumoniae* | R |  | R | R |  | R |  |
| 285 | Urine | *Klebsiella pneumoniae* | R |  | R | R |  |  |  |
| 286 | Urine | *Klebsiella pneumoniae* | R |  | R | R |  |  |  |
| 287 | Urine | *Klebsiella pneumoniae* | R |  | R |  | R | R |  |
| 288 | Urine | *Klebsiella pneumoniae* | R |  | R |  | R |  |  |
| 289 | Urine | *Klebsiella pneumoniae* | R |  | R |  | R | R |  |
| 290 | Urine | *Klebsiella pneumoniae* | R | R | R |  | R | R |  |
| 291 | Urine | *Klebsiella pneumoniae* | R |  | R |  | R |  |  |
| 292 | Urine | *Klebsiella pneumoniae* | R |  | R |  | R |  |  |
| 293 | Urine | *Klebsiella pneumoniae* | R | R |  | R |  |  |  |
| 294 | Urine | *Klebsiella pneumoniae* | S | R |  |  | S |  |  |
| 295 | Urine | *Klebsiella pneumoniae* | R | R |  | R |  | R |  |
| 296 | Urine | *Klebsiella pneumoniae* | R | R |  | R |  |  |  |
| 297 | Urine | *Klebsiella pneumoniae* | R |  | R |  | R |  |  |
| 298 | Urine | *Klebsiella pneumoniae* | R | R |  | R | R |  |  |
| 299 | Urine | *Klebsiella pneumoniae* | R | R |  | R |  | R |  |
| 300 | Blood | *Staphylococcus aureus* |  |  |  |  |  |  | R |
| 301 | Blood | *Staphylococcus aureus* |  | R |  |  | R |  |  |
| 302 | Blood | *Staphylococcus aureus* |  | R |  |  |  |  |  |
| 303 | Blood | *Staphylococcus aureus* |  | R |  |  |  |  |  |
| 304 | Blood | *Staphylococcus aureus* |  |  |  |  |  |  |  |
| 305 | Blood | *Staphylococcus aureus* |  |  |  |  |  |  |  |
| 306 | Blood | *Staphylococcus aureus* |  |  |  |  |  |  |  |
| 307 | Blood | *Staphylococcus aureus* | S | R |  | R |  | R |  |
| 308 | Blood | *Staphylococcus aureus* | R |  |  |  |  |  |  |
| 309 | Vaginal swabs | *Staphylococcus aureus* |  |  |  |  |  |  |  |
| 310 | Vaginal swabs | *Staphylococcus aureus* |  |  |  |  |  |  |  |
| 311 | Vaginal swabs | *Staphylococcus aureus* |  |  |  |  |  |  |  |
| 312 | Pus | *Staphylococcus aureus* |  |  |  |  |  |  | R |
| 313 | Pus | *Staphylococcus aureus* | R |  |  | R |  |  |  |
| 314 | Pus | *Staphylococcus aureus* |  |  |  |  |  |  |  |
| 315 | Pus | *Staphylococcus aureus* | R |  |  | R |  |  |  |
| 316 | Pus | *Staphylococcus aureus* |  |  |  |  |  |  | R |
| 317 | Pus | *Staphylococcus aureus* |  |  |  |  |  |  | R |
| 318 | Pus | *Staphylococcus aureus* |  |  |  |  |  | R |  |
| 319 | Pus | *Staphylococcus aureus* |  |  |  |  |  |  | R |
| 320 | Urine | *Staphylococcus aureus* |  |  |  |  |  | S | R |
| 321 | Urine | *Staphylococcus aureus* | R | R |  | R |  | R |  |
| 322 | Urine | *Staphylococcus aureus* |  | R |  |  |  |  | R |

Antimicrobial susceptibility pattern was performed by disk diffusion method and results were interpreted according to CLSI guideline 2022. Here we present the sensitivity results of most prevalent isolates on selected antimicrobials with higher resistance rates. The empty cells means that isolate was not tested against that antimicrobial, either due to testing panel does not include such antimicrobial as different antimicrobials are tested against each isolate depending on which sample it comes from (CLSI guideline) or due to unavailability of the disk on the testing time day. The inhibition zone was determined and results were recorded as sensitive or resistant according to CLSI guideline. AMC: Amoxicillin clavulanic acid; AMP: ampicillin; CAZ: ceftazidime; CRO: ceftriaxone; CTX: cefotaxime; CXM: cefuroxime; DO: doxycycline; R: resistance; S: sensitive or susceptible.
